# (Naphthalene-2,3-diolato-κ^2^ *O*,*O*′)[tris­(2-pyridyl­meth­yl)amine-κ^4^ *N*]cobalt(III) hexa­fluoridophosphate hemihydrate

**DOI:** 10.1107/S1600536811023270

**Published:** 2011-06-25

**Authors:** Yan-Hua Guo, Yu-Min Zhang, Ai-Hua Li, Fan Yu

**Affiliations:** a School of Chemistry and Environmental Engineering, Jianghan University, Wuhan, Hubei 430056, People’s Republic of China

## Abstract

In the title complex, [Co(C_10_H_6_O_2_)(C_18_H_18_N_4_)]PF_6_·0.5H_2_O, the Co^III^ ion is six-coordinated in a distorted octa­hedral geometry by four N atoms from a tris­(2-pyridyl­meth­yl)amine ligand and two O atoms from a naphthalene-2,3-diolate ligand. The asymmetric unit contains two complex cations, two hexa­fluoridophosphate anions and one uncoordinated water mol­ecule. In one of the hexa­fluoridophosphate anions, four of the F aroms are disordered over two sets of sites in a 0.632 (11):0.368 (11) ratio. In the crystal, the cations, anions and water mol­ecules are connected by O—H⋯O and O—H⋯F hydrogen bonds. π–π inter­actions are present between the pyridine rings [centroid–centroid distance = 3.814 (1) Å].

## Related literature

For related structures, see: Caneschi *et al.* (2001 ▶); Tao *et al.* (2006 ▶); Tinoco *et al.* (2008 ▶). For the octa­hedral distortion parameter, see: Li *et al.* (2010 ▶).
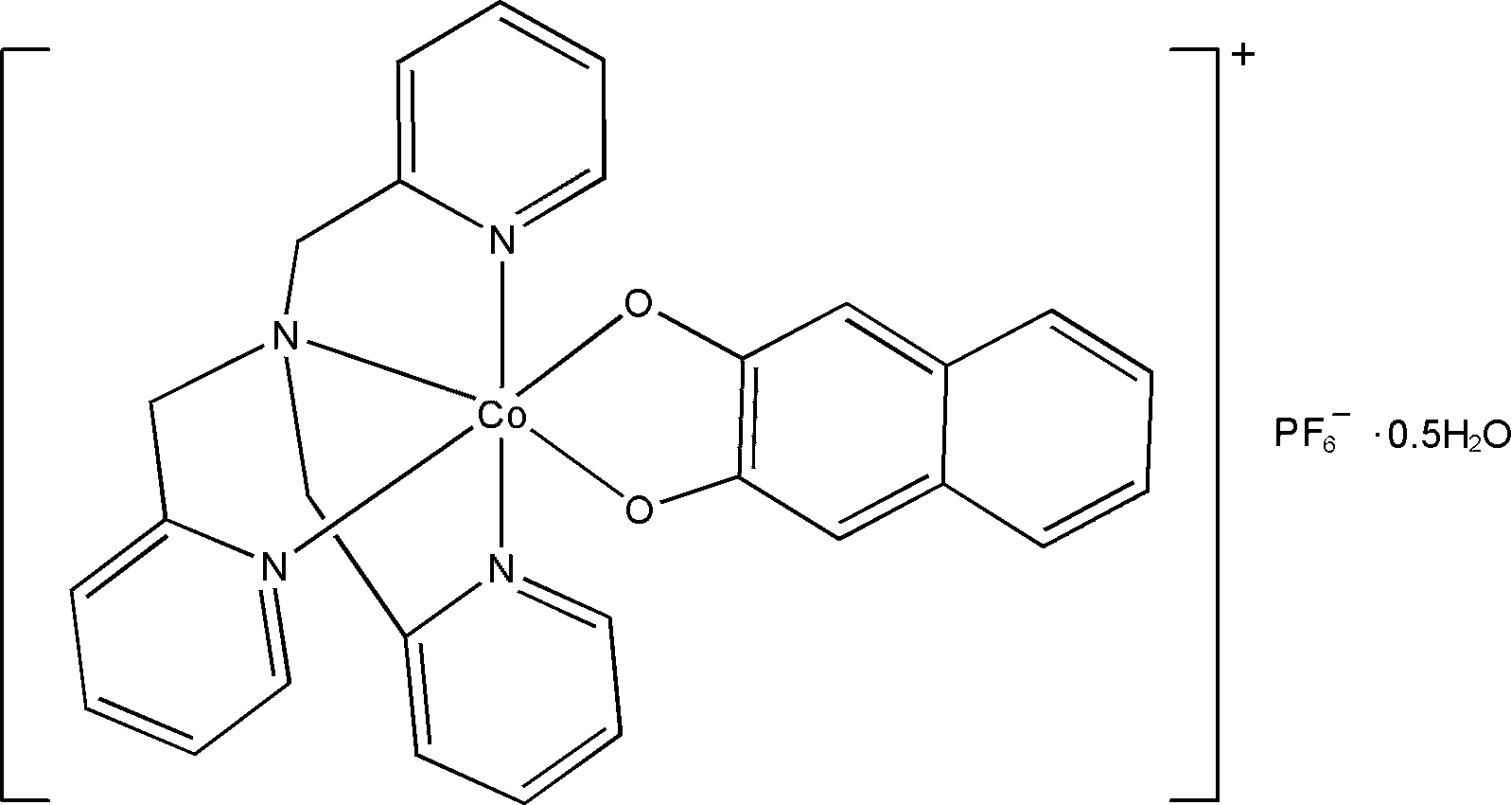


## Experimental

### 

#### Crystal data

[Co(C_10_H_6_O_2_)(C_18_H_18_N_4_)]PF_6_·0.5H_2_O
*M* *_r_* = 661.42
Triclinic, 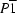

*a* = 10.9879 (3) Å
*b* = 13.4543 (3) Å
*c* = 18.7518 (6) Å
α = 86.250 (2)°
β = 74.506 (3)°
γ = 86.648 (2)°
*V* = 2663.28 (13) Å^3^
*Z* = 4
Mo *K*α radiation
μ = 0.79 mm^−1^
*T* = 293 K
0.30 × 0.20 × 0.20 mm

#### Data collection

Oxford Diffraction Gemini S Ultra CCD diffractometer
Absorption correction: multi-scan (*CrysAlis RED*; Oxford Diffraction, 2006 ▶) *T*_min_ = 0.828, *T*_max_ = 0.855
19595 measured reflections
9170 independent reflections
7198 reflections with *I* > 2σ(*I*)
*R*_int_ = 0.018

#### Refinement

*R*[*F* ^2^ > 2σ(*F* ^2^)] = 0.036
*wR*(*F* ^2^) = 0.098
*S* = 1.10
9170 reflections
803 parameters
2 restraints
H-atom parameters constrained
Δρ_max_ = 0.71 e Å^−3^
Δρ_min_ = −0.72 e Å^−3^

### 

Data collection: *CrysAlis CCD* (Oxford Diffraction, 2006 ▶); cell refinement: *CrysAlis RED* (Oxford Diffraction, 2006 ▶); data reduction: *CrysAlis RED*; program(s) used to solve structure: *SHELXS97* (Sheldrick, 2008 ▶); program(s) used to refine structure: *SHELXL97* (Sheldrick, 2008 ▶); molecular graphics: *SHELXTL* (Sheldrick, 2008 ▶); software used to prepare material for publication: *SHELXTL*.

## Supplementary Material

Crystal structure: contains datablock(s) I, global. DOI: 10.1107/S1600536811023270/hy2438sup1.cif

Structure factors: contains datablock(s) I. DOI: 10.1107/S1600536811023270/hy2438Isup2.hkl

Additional supplementary materials:  crystallographic information; 3D view; checkCIF report

## Figures and Tables

**Table 1:** Hydrogen-bond geometry (Å, °)

*D*—H⋯*A*	*D*—H	H⋯*A*	*D*⋯*A*	*D*—H⋯*A*
O1*W*—H1*WC*⋯O3	0.85	1.97	2.808 (3)	170
O1*W*—H1*WD*⋯F4	0.85	2.11	2.940 (5)	165
O1*W*—H1*WD*⋯F4*A*	0.85	2.55	3.194 (11)	133
O1*W*—H1*WD*⋯F6*A*	0.85	2.30	3.142 (11)	171

**Fig. 1.**
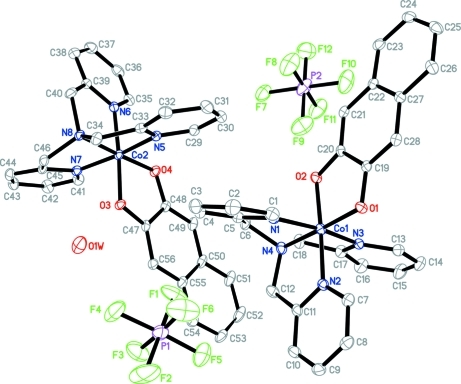
Molecular structure of the title compound, showing 30% probability displacement ellipsoids. H atoms and minor disordered positions of the F atoms have been omitted for clarity.

## supplementary crystallographic information

### Comment

Coordination of catecholates (cat) to transition-metal ions, for example, cobalt or iron, as bidentate chelate ligands tends to stabilize the metal ions in high oxidation states, and may result in functional complexes with interesting properties (Tao *et al.*, 2006). Searching for new types of catecholates to construct more functional materials still poses a great challenge. Naphthalene-2,3-diol, as one kind of catecholates, possesses the bidentate chelate mode and much stronger π-conjugate systems. Complexes of some transitional metals containing naphthalene-2,3-diol have been synthesized and crystallographically characterized (Tinoco *et al.*, 2008), but no such comlpexes of cobalt have been documented since the potential valence tautomerism might be exhibited in the system of [Co(N4)(cat)]*X* (N4 = tripodal ligands containing four coordinated N atoms, *X* = PF_6_^-^ or BPh_4_^-^) (Caneschi *et al.*, 2001). In this study, a new mononuclear Co(III) compound with the tripodal ligand tris(2-pyridylmethyl)amine (tpa), naphthalene-2,3-diolate (ND) and counteranions PF_6_ has been prepared and structurally characterized. The present study is the first crystal structure determination of a cobalt(III) complex with ND.

The molecular structure of the title compound is shown in Fig. 1. The asymmetric unit contains two complex cations, two hexafluoridophosphate anions and one lattice water molecule. Each Co^III^ ion is six-coordinated in a distorted octahedral geometry by four N atoms from a tpa ligand and two O atoms from a bidentate chelate ND ligand. The equatorial plane is formed by two O atoms of ND and two N atoms from tpa (one is the tri-bridged aliphatic N atom and the other is from one pyridine ring). The axial positions are occupied by the other two pyridine N atoms. The coordination features of the two Co atoms are fully consistent with the formulation of a Co^III^–catecholato complex. The Co—N [average 1.927 (2) and 1.931 (2) Å for Co1 and Co2] and Co—O distances [average 1.882 (2) and 1.885 (2) Å for Co1 and Co2] are in agreement with those values usually observed in the other Co^III^–catecholato complexes (Caneschi *et al.*, 2001).

To compare the distorted octahedra of Co1 and Co2 atoms, the parameter σ (sum of the deviations of *cis* N—Co—N angles from 90°) that can be commonly quantified by an octahedral distortion parameter was introduced (Li *et al.*, 2010). In the present case, the values are 31.4° and 38.1° for Co1 and Co2, implying low-spin states at room temperature, as confirmed by magnetic characterization. Variable temperature magnetic measurements of the title compound in the region of 2–380 K show the obvious diamagnetism over the temperature observed, indicating the absence of valence tautomeric transiton.

### Experimental

To a well stirred methanol solution (20 ml) containing tpa (2.02 mmol) and CoCl_2_.6H_2_O (2.0 mmol) was added a methanol solution (10 ml) containing ND (0.5 mmol) and triethylamine (140 µl) under inert atmosphere. The resulting mixture was gently stirred at room temperature for 2 h and then KPF_6_ (1 mmol) was added. The solution was stirred for several minutes and then filtered. Green crystals of the title compound were obtained by slow evaporation of the filtrate.

### Refinement

H atoms on C atoms were placed geometrically and refined using a riding model, with C—H = 0.93 (aromatic) and 0.97 (CH_2_) Å and *U*_iso_(H) = 1.2*U*_eq_(C). Water H atoms were located in a difference Fourier map and refined as riding atoms, with O—H = 0.85 Å and *U*_iso_(H) = 1.2*U*_eq_(O). One of the PF_6_^-^ anions is obviously 2-fold disordered and thus was modeled with four of the F atoms over two sites in a 0.632 (11):0.368 (11) ratio.

### Crystal data

**Table:**

[Co(C_10_H_6_O_2_)(C_18_H_18_N_4_)]PF_6_·0.5H_2_O	*Z* = 4
*M**_r_* = 661.42	*F*(000) = 1348
Triclinic, *P*1	*D*_x_ = 1.650 Mg m^−^^3^
Hall symbol: -P 1	Mo *K*α radiation, λ = 0.71073 Å
*a* = 10.9879 (3) Å	Cell parameters from 12113 reflections
*b* = 13.4543 (3) Å	θ = 2.3–29.1°
*c* = 18.7518 (6) Å	µ = 0.79 mm^−^^1^
α = 86.250 (2)°	*T* = 293 K
β = 74.506 (3)°	Block, green
γ = 86.648 (2)°	0.30 × 0.20 × 0.20 mm
*V* = 2663.28 (13) Å^3^	

### Data collection

**Table:**

Oxford Diffraction Gemini S Ultra CCD diffractometer	9170 independent reflections
Radiation source: fine-focus sealed tube	7198 reflections with *I* > 2σ(*I*)
graphite	*R*_int_ = 0.018
ω scans	θ_max_ = 25.0°, θ_min_ = 2.3°
Absorption correction: multi-scan (*CrysAlis RED*; Oxford Diffraction, 2006)	*h* = −13→12
*T*_min_ = 0.828, *T*_max_ = 0.855	*k* = −15→15
19595 measured reflections	*l* = −22→19

### Refinement

**Table:**

Refinement on *F*^2^	Primary atom site location: structure-invariant direct methods
Least-squares matrix: full	Secondary atom site location: difference Fourier map
*R*[*F*^2^ > 2σ(*F*^2^)] = 0.036	Hydrogen site location: inferred from neighbouring sites
*wR*(*F*^2^) = 0.098	H-atom parameters constrained
*S* = 1.10	*w* = 1/[σ^2^(*F*_o_^2^) + (0.0576*P*)^2^] where *P* = (*F*_o_^2^ + 2*F*_c_^2^)/3
9170 reflections	(Δ/σ)_max_ = 0.003
803 parameters	Δρ_max_ = 0.71 e Å^−^^3^
2 restraints	Δρ_min_ = −0.72 e Å^−^^3^

### Fractional atomic coordinates and isotropic or equivalent isotropic displacement parameters (Å^2^)

**Table:**

	*x*	*y*	*z*	*U*_iso_*/*U*_eq_	Occ. (<1)
Co1	0.58899 (3)	0.46680 (2)	0.318907 (17)	0.02037 (10)	
Co2	0.39509 (3)	−0.02204 (2)	0.188627 (18)	0.02001 (10)	
N2	0.73644 (17)	0.45602 (14)	0.35507 (11)	0.0224 (4)	
O4	0.47568 (15)	0.06734 (12)	0.11187 (9)	0.0251 (4)	
O3	0.54083 (14)	−0.02494 (11)	0.22443 (9)	0.0226 (4)	
O2	0.44824 (14)	0.47143 (11)	0.28042 (9)	0.0230 (4)	
N4	0.67728 (18)	0.37566 (14)	0.24391 (11)	0.0252 (5)	
N5	0.31832 (17)	0.08058 (14)	0.25503 (11)	0.0227 (4)	
N6	0.25112 (17)	−0.02837 (14)	0.14947 (11)	0.0233 (5)	
N1	0.52979 (17)	0.34792 (14)	0.37680 (11)	0.0237 (5)	
O1	0.49857 (14)	0.55475 (11)	0.39087 (9)	0.0220 (4)	
N3	0.66662 (17)	0.56966 (14)	0.24767 (11)	0.0223 (4)	
N8	0.30731 (17)	−0.11206 (14)	0.26909 (11)	0.0211 (4)	
C47	0.6272 (2)	0.03462 (16)	0.17946 (13)	0.0217 (5)	
N7	0.46057 (17)	−0.14099 (14)	0.13738 (11)	0.0222 (4)	
C5	0.5426 (2)	0.26689 (18)	0.33596 (14)	0.0255 (6)	
C45	0.4380 (2)	−0.22517 (17)	0.18101 (13)	0.0215 (5)	
C6	0.5935 (2)	0.28873 (18)	0.25442 (14)	0.0293 (6)	
H6A	0.5246	0.3047	0.2317	0.035*	
H6B	0.6414	0.2310	0.2314	0.035*	
C20	0.3554 (2)	0.53030 (16)	0.31991 (13)	0.0199 (5)	
C33	0.2732 (2)	0.04687 (18)	0.32610 (14)	0.0258 (6)	
C19	0.3831 (2)	0.57717 (16)	0.38044 (13)	0.0197 (5)	
C46	0.3859 (2)	−0.20752 (17)	0.26181 (13)	0.0228 (5)	
H46A	0.3345	−0.2624	0.2864	0.027*	
H46B	0.4544	−0.2031	0.2849	0.027*	
C11	0.8293 (2)	0.39339 (17)	0.31851 (14)	0.0265 (6)	
P1	0.84409 (9)	0.05999 (7)	0.40007 (6)	0.0547 (3)	
P2	0.21255 (9)	0.48771 (7)	0.09115 (5)	0.0522 (2)	
C44	0.4640 (2)	−0.31767 (18)	0.15167 (14)	0.0274 (6)	
H44A	0.4442	−0.3752	0.1820	0.033*	
C27	0.1773 (2)	0.66391 (17)	0.40468 (13)	0.0218 (5)	
C48	0.5890 (2)	0.08879 (17)	0.12045 (13)	0.0240 (5)	
C22	0.1504 (2)	0.61779 (17)	0.34397 (14)	0.0230 (5)	
C10	0.9396 (2)	0.37685 (19)	0.33954 (17)	0.0348 (7)	
H10A	1.0032	0.3342	0.3135	0.042*	
C28	0.2948 (2)	0.64224 (16)	0.42101 (13)	0.0217 (5)	
H28A	0.3124	0.6727	0.4600	0.026*	
C1	0.4884 (2)	0.33829 (19)	0.45056 (14)	0.0290 (6)	
H1A	0.4826	0.3941	0.4782	0.035*	
C4	0.5107 (2)	0.17442 (19)	0.36866 (16)	0.0331 (6)	
H4A	0.5195	0.1193	0.3400	0.040*	
C40	0.1767 (2)	−0.1271 (2)	0.26179 (15)	0.0308 (6)	
H40A	0.1586	−0.1972	0.2708	0.037*	
H40B	0.1153	−0.0901	0.2989	0.037*	
C21	0.2425 (2)	0.54933 (17)	0.30340 (13)	0.0232 (5)	
H21A	0.2255	0.5170	0.2650	0.028*	
C26	0.0850 (2)	0.73278 (18)	0.44416 (14)	0.0298 (6)	
H26A	0.0996	0.7629	0.4842	0.036*	
C29	0.3077 (2)	0.17830 (18)	0.23696 (15)	0.0288 (6)	
H29A	0.3378	0.2010	0.1878	0.035*	
F7	0.23669 (17)	0.37936 (14)	0.05936 (11)	0.0616 (6)	
C42	0.5472 (2)	−0.23759 (19)	0.03302 (15)	0.0334 (6)	
H42A	0.5878	−0.2405	−0.0172	0.040*	
C39	0.1645 (2)	−0.09336 (17)	0.18731 (15)	0.0267 (6)	
C7	0.7522 (2)	0.50248 (18)	0.41344 (14)	0.0267 (6)	
H7A	0.6882	0.5459	0.4383	0.032*	
C43	0.5197 (2)	−0.32392 (19)	0.07678 (15)	0.0320 (6)	
H43A	0.5384	−0.3858	0.0561	0.038*	
C8	0.8598 (2)	0.48730 (19)	0.43693 (15)	0.0317 (6)	
H8A	0.8684	0.5194	0.4777	0.038*	
C50	0.7857 (2)	0.17544 (18)	0.08879 (14)	0.0290 (6)	
F8	0.1210 (2)	0.44372 (17)	0.16590 (12)	0.0861 (8)	
C34	0.2983 (2)	−0.06308 (17)	0.33942 (13)	0.0260 (6)	
H34A	0.3766	−0.0745	0.3538	0.031*	
H34B	0.2301	−0.0902	0.3788	0.031*	
C24	−0.0491 (2)	0.71198 (19)	0.36415 (16)	0.0339 (6)	
H24A	−0.1235	0.7291	0.3507	0.041*	
C13	0.6821 (2)	0.66416 (18)	0.26061 (14)	0.0267 (6)	
H13A	0.6462	0.6871	0.3077	0.032*	
C49	0.6659 (2)	0.15840 (17)	0.07697 (14)	0.0272 (6)	
H49A	0.6397	0.1948	0.0394	0.033*	
F1	0.7632 (2)	0.09795 (18)	0.34540 (14)	0.0890 (8)	
C32	0.2138 (2)	0.11021 (19)	0.38085 (16)	0.0339 (6)	
H32A	0.1805	0.0857	0.4293	0.041*	
C35	0.2441 (2)	0.00968 (18)	0.08276 (14)	0.0271 (6)	
H35A	0.3027	0.0554	0.0573	0.032*	
C55	0.8259 (2)	0.11851 (18)	0.14502 (15)	0.0289 (6)	
C23	0.0365 (2)	0.64413 (18)	0.32513 (15)	0.0298 (6)	
H23A	0.0192	0.6149	0.2855	0.036*	
C38	0.0709 (2)	−0.1228 (2)	0.15771 (17)	0.0351 (7)	
H38A	0.0124	−0.1681	0.1840	0.042*	
C41	0.5137 (2)	−0.14674 (19)	0.06448 (14)	0.0294 (6)	
H41A	0.5282	−0.0884	0.0346	0.035*	
O1W	0.61242 (18)	−0.12299 (14)	0.34471 (11)	0.0455 (5)	
H1WC	0.5953	−0.0871	0.3093	0.055*	
H1WD	0.6526	−0.0886	0.3666	0.055*	
C56	0.7445 (2)	0.04671 (17)	0.18992 (14)	0.0255 (6)	
H56A	0.7709	0.0079	0.2264	0.031*	
C17	0.7200 (2)	0.53593 (19)	0.17929 (14)	0.0279 (6)	
C18	0.6890 (2)	0.43102 (18)	0.17088 (14)	0.0311 (6)	
H18A	0.6102	0.4305	0.1569	0.037*	
H18B	0.7554	0.4001	0.1328	0.037*	
F12	0.09752 (19)	0.50702 (17)	0.05506 (13)	0.0790 (7)	
C36	0.1521 (2)	−0.01724 (19)	0.05096 (16)	0.0338 (6)	
H36A	0.1490	0.0096	0.0045	0.041*	
F11	0.3049 (2)	0.53198 (16)	0.01627 (11)	0.0783 (7)	
C30	0.2529 (2)	0.24604 (19)	0.28993 (17)	0.0347 (7)	
H30A	0.2485	0.3139	0.2771	0.042*	
C52	0.9773 (3)	0.2669 (2)	0.06041 (18)	0.0459 (9)	
H52A	1.0277	0.3164	0.0327	0.055*	
F10	0.1905 (2)	0.59533 (16)	0.12268 (13)	0.0904 (8)	
C14	0.7498 (2)	0.7284 (2)	0.20608 (16)	0.0351 (7)	
H14A	0.7571	0.7944	0.2158	0.042*	
C53	1.0167 (2)	0.2111 (2)	0.11599 (19)	0.0437 (8)	
H53A	1.0936	0.2235	0.1248	0.052*	
C51	0.8643 (3)	0.24928 (19)	0.04626 (16)	0.0383 (7)	
H51A	0.8394	0.2861	0.0085	0.046*	
C16	0.7927 (3)	0.5955 (2)	0.12338 (15)	0.0380 (7)	
H16A	0.8317	0.5703	0.0774	0.046*	
F9	0.3310 (2)	0.46932 (18)	0.12566 (13)	0.0881 (7)	
C31	0.2048 (2)	0.2107 (2)	0.36226 (17)	0.0375 (7)	
H31A	0.1662	0.2548	0.3986	0.045*	
C2	0.4539 (2)	0.2475 (2)	0.48682 (16)	0.0372 (7)	
H2A	0.4237	0.2421	0.5382	0.045*	
C12	0.8044 (2)	0.3440 (2)	0.25510 (16)	0.0354 (7)	
H12A	0.8085	0.2723	0.2643	0.042*	
H12B	0.8694	0.3606	0.2103	0.042*	
C25	−0.0245 (2)	0.75565 (19)	0.42466 (16)	0.0349 (7)	
H25A	−0.0837	0.8007	0.4517	0.042*	
C9	0.9558 (2)	0.42358 (19)	0.39928 (17)	0.0358 (7)	
H9A	1.0300	0.4126	0.4142	0.043*	
C54	0.9438 (2)	0.13818 (19)	0.15787 (17)	0.0361 (7)	
H54A	0.9714	0.1015	0.1948	0.043*	
C37	0.0653 (3)	−0.0842 (2)	0.08885 (17)	0.0387 (7)	
H37A	0.0030	−0.1035	0.0681	0.046*	
C15	0.8066 (3)	0.6935 (2)	0.13685 (16)	0.0427 (8)	
H15A	0.8537	0.7355	0.0996	0.051*	
C3	0.4651 (2)	0.1644 (2)	0.44508 (17)	0.0375 (7)	
H3A	0.4423	0.1024	0.4682	0.045*	
F2	0.9295 (3)	0.0197 (3)	0.4509 (2)	0.1549 (16)	
F3	0.9293 (6)	−0.0004 (5)	0.3442 (3)	0.0836 (19)	0.632 (11)
F4	0.7640 (5)	−0.0405 (6)	0.4326 (3)	0.0800 (18)	0.632 (11)
F5	0.9197 (6)	0.1536 (4)	0.3817 (5)	0.099 (2)	0.632 (11)
F6	0.7441 (5)	0.1175 (6)	0.4690 (3)	0.106 (3)	0.632 (11)
F3A	0.9775 (10)	0.1049 (11)	0.3294 (7)	0.117 (6)	0.368 (11)
F5A	0.830 (2)	0.1525 (8)	0.4298 (8)	0.196 (8)	0.368 (11)
F6A	0.7294 (9)	0.0143 (8)	0.4362 (6)	0.094 (3)	0.368 (11)
F4A	0.8800 (10)	−0.0335 (7)	0.3340 (6)	0.076 (3)	0.368 (11)

### Atomic displacement parameters (Å^2^)

**Table:**

	*U*^11^	*U*^22^	*U*^33^	*U*^12^	*U*^13^	*U*^23^
Co1	0.02166 (17)	0.02106 (18)	0.01703 (18)	0.00139 (13)	−0.00264 (13)	−0.00355 (13)
Co2	0.02021 (17)	0.02004 (18)	0.01921 (18)	−0.00463 (13)	−0.00466 (13)	0.00419 (13)
N2	0.0226 (10)	0.0203 (10)	0.0229 (11)	−0.0004 (8)	−0.0043 (8)	0.0022 (9)
O4	0.0263 (9)	0.0249 (9)	0.0243 (9)	−0.0081 (7)	−0.0080 (7)	0.0083 (7)
O3	0.0207 (8)	0.0246 (9)	0.0221 (9)	−0.0067 (7)	−0.0058 (7)	0.0060 (7)
O2	0.0246 (8)	0.0243 (9)	0.0203 (9)	0.0029 (7)	−0.0059 (7)	−0.0076 (7)
N4	0.0278 (11)	0.0221 (11)	0.0241 (11)	0.0011 (8)	−0.0032 (9)	−0.0055 (9)
N5	0.0200 (10)	0.0209 (11)	0.0278 (12)	−0.0028 (8)	−0.0077 (9)	0.0015 (9)
N6	0.0243 (10)	0.0197 (10)	0.0267 (12)	0.0000 (8)	−0.0083 (9)	−0.0013 (9)
N1	0.0214 (10)	0.0241 (11)	0.0246 (11)	0.0007 (8)	−0.0047 (8)	−0.0016 (9)
O1	0.0230 (8)	0.0253 (9)	0.0180 (9)	0.0043 (7)	−0.0062 (7)	−0.0051 (7)
N3	0.0215 (10)	0.0212 (11)	0.0245 (11)	0.0007 (8)	−0.0064 (8)	−0.0035 (9)
N8	0.0198 (10)	0.0211 (10)	0.0217 (11)	−0.0056 (8)	−0.0043 (8)	0.0035 (9)
C47	0.0253 (12)	0.0159 (12)	0.0212 (13)	−0.0031 (10)	−0.0008 (10)	−0.0029 (10)
N7	0.0210 (10)	0.0243 (11)	0.0209 (11)	−0.0037 (8)	−0.0051 (8)	0.0034 (9)
C5	0.0211 (12)	0.0249 (13)	0.0301 (14)	−0.0003 (10)	−0.0056 (10)	−0.0035 (11)
C45	0.0206 (12)	0.0230 (13)	0.0202 (13)	−0.0048 (10)	−0.0048 (10)	0.0044 (10)
C6	0.0338 (14)	0.0224 (13)	0.0301 (15)	−0.0029 (11)	−0.0033 (11)	−0.0095 (11)
C20	0.0250 (12)	0.0156 (12)	0.0166 (12)	−0.0027 (9)	−0.0014 (10)	0.0020 (9)
C33	0.0186 (12)	0.0285 (14)	0.0299 (15)	−0.0030 (10)	−0.0053 (10)	−0.0015 (11)
C19	0.0220 (12)	0.0180 (12)	0.0176 (12)	−0.0030 (9)	−0.0032 (9)	0.0038 (10)
C46	0.0255 (12)	0.0197 (12)	0.0221 (13)	−0.0046 (10)	−0.0051 (10)	0.0051 (10)
C11	0.0246 (13)	0.0194 (13)	0.0309 (15)	−0.0008 (10)	−0.0007 (11)	0.0050 (11)
P1	0.0571 (5)	0.0473 (5)	0.0745 (7)	−0.0099 (4)	−0.0443 (5)	0.0085 (5)
P2	0.0623 (6)	0.0608 (6)	0.0336 (4)	−0.0258 (5)	−0.0052 (4)	−0.0133 (4)
C44	0.0326 (14)	0.0233 (13)	0.0262 (14)	−0.0040 (11)	−0.0082 (11)	0.0041 (11)
C27	0.0218 (12)	0.0203 (12)	0.0195 (13)	−0.0015 (10)	−0.0006 (10)	0.0066 (10)
C48	0.0253 (13)	0.0220 (13)	0.0225 (13)	−0.0038 (10)	−0.0011 (10)	−0.0036 (11)
C22	0.0225 (12)	0.0181 (12)	0.0255 (13)	−0.0054 (10)	−0.0024 (10)	0.0071 (10)
C10	0.0237 (13)	0.0279 (14)	0.0486 (18)	0.0026 (11)	−0.0042 (12)	0.0038 (13)
C28	0.0279 (12)	0.0212 (12)	0.0151 (12)	−0.0045 (10)	−0.0037 (10)	0.0006 (10)
C1	0.0283 (13)	0.0325 (15)	0.0244 (14)	0.0001 (11)	−0.0043 (11)	−0.0009 (12)
C4	0.0318 (14)	0.0248 (14)	0.0436 (17)	−0.0009 (11)	−0.0116 (12)	−0.0030 (12)
C40	0.0207 (12)	0.0326 (14)	0.0377 (16)	−0.0105 (11)	−0.0055 (11)	0.0070 (12)
C21	0.0264 (12)	0.0200 (12)	0.0235 (13)	−0.0070 (10)	−0.0064 (10)	0.0008 (10)
C26	0.0328 (14)	0.0255 (13)	0.0254 (14)	0.0022 (11)	0.0006 (11)	0.0018 (11)
C29	0.0254 (13)	0.0243 (14)	0.0367 (16)	−0.0040 (10)	−0.0092 (11)	0.0050 (12)
F7	0.0586 (11)	0.0632 (12)	0.0591 (13)	−0.0198 (9)	−0.0014 (10)	−0.0195 (10)
C42	0.0411 (15)	0.0379 (16)	0.0189 (13)	0.0003 (12)	−0.0047 (11)	0.0005 (12)
C39	0.0215 (12)	0.0212 (13)	0.0370 (15)	0.0000 (10)	−0.0070 (11)	−0.0021 (11)
C7	0.0289 (13)	0.0266 (13)	0.0234 (14)	−0.0046 (10)	−0.0049 (11)	0.0021 (11)
C43	0.0373 (14)	0.0270 (14)	0.0311 (15)	0.0009 (11)	−0.0076 (12)	−0.0046 (12)
C8	0.0368 (15)	0.0313 (15)	0.0294 (15)	−0.0118 (12)	−0.0133 (12)	0.0091 (12)
C50	0.0310 (14)	0.0243 (13)	0.0257 (14)	−0.0094 (11)	0.0071 (11)	−0.0106 (11)
F8	0.1022 (17)	0.0943 (17)	0.0471 (13)	−0.0415 (14)	0.0154 (12)	−0.0112 (12)
C34	0.0283 (13)	0.0279 (13)	0.0187 (13)	−0.0056 (10)	−0.0012 (10)	0.0030 (11)
C24	0.0223 (13)	0.0298 (14)	0.0458 (18)	−0.0041 (11)	−0.0053 (12)	0.0129 (13)
C13	0.0259 (13)	0.0295 (14)	0.0245 (14)	−0.0003 (11)	−0.0063 (11)	−0.0034 (11)
C49	0.0371 (14)	0.0231 (13)	0.0184 (13)	−0.0074 (11)	−0.0015 (11)	0.0010 (10)
F1	0.0809 (15)	0.1205 (19)	0.0817 (17)	0.0555 (13)	−0.0542 (13)	−0.0374 (15)
C32	0.0263 (13)	0.0371 (16)	0.0347 (16)	−0.0007 (11)	−0.0016 (11)	−0.0029 (13)
C35	0.0307 (13)	0.0248 (13)	0.0257 (14)	0.0046 (10)	−0.0087 (11)	−0.0014 (11)
C55	0.0231 (13)	0.0233 (13)	0.0353 (16)	−0.0034 (10)	0.0046 (11)	−0.0141 (12)
C23	0.0259 (13)	0.0269 (14)	0.0372 (16)	−0.0085 (11)	−0.0102 (11)	0.0086 (12)
C38	0.0275 (14)	0.0304 (15)	0.0505 (19)	−0.0027 (11)	−0.0144 (13)	−0.0052 (14)
C41	0.0321 (14)	0.0327 (14)	0.0201 (13)	−0.0034 (11)	−0.0027 (11)	0.0057 (11)
O1W	0.0623 (13)	0.0432 (12)	0.0392 (12)	−0.0188 (10)	−0.0279 (11)	0.0132 (10)
C56	0.0232 (12)	0.0213 (13)	0.0301 (14)	−0.0011 (10)	−0.0034 (11)	−0.0039 (11)
C17	0.0306 (13)	0.0318 (14)	0.0197 (13)	−0.0012 (11)	−0.0030 (11)	−0.0049 (11)
C18	0.0399 (15)	0.0285 (14)	0.0207 (13)	−0.0020 (11)	0.0004 (11)	−0.0060 (11)
F12	0.0681 (13)	0.0928 (16)	0.0812 (17)	0.0023 (11)	−0.0236 (12)	−0.0309 (14)
C36	0.0393 (15)	0.0348 (15)	0.0318 (16)	0.0115 (12)	−0.0180 (13)	−0.0094 (13)
F11	0.0886 (15)	0.0851 (15)	0.0521 (13)	−0.0394 (12)	0.0049 (11)	−0.0031 (12)
C30	0.0278 (13)	0.0242 (14)	0.0522 (19)	0.0018 (11)	−0.0114 (13)	−0.0013 (13)
C52	0.0400 (17)	0.0394 (17)	0.0459 (19)	−0.0214 (14)	0.0185 (14)	−0.0202 (16)
F10	0.125 (2)	0.0723 (15)	0.0735 (16)	−0.0271 (13)	−0.0134 (14)	−0.0357 (13)
C14	0.0380 (15)	0.0301 (15)	0.0366 (16)	−0.0122 (12)	−0.0065 (13)	−0.0019 (13)
C53	0.0224 (14)	0.0376 (17)	0.064 (2)	−0.0100 (12)	0.0081 (14)	−0.0245 (17)
C51	0.0450 (16)	0.0310 (15)	0.0307 (15)	−0.0153 (13)	0.0088 (13)	−0.0096 (13)
C16	0.0384 (15)	0.0454 (17)	0.0244 (15)	−0.0084 (13)	0.0041 (12)	−0.0063 (13)
F9	0.0897 (16)	0.1142 (19)	0.0770 (17)	−0.0291 (14)	−0.0437 (14)	−0.0128 (15)
C31	0.0312 (14)	0.0338 (16)	0.0451 (18)	0.0064 (12)	−0.0055 (13)	−0.0108 (14)
C2	0.0373 (15)	0.0427 (17)	0.0285 (15)	−0.0049 (13)	−0.0052 (12)	0.0079 (13)
C12	0.0273 (13)	0.0330 (15)	0.0416 (17)	0.0079 (11)	−0.0018 (12)	−0.0093 (13)
C25	0.0262 (14)	0.0298 (14)	0.0386 (17)	0.0048 (11)	0.0053 (12)	0.0076 (13)
C9	0.0279 (14)	0.0305 (15)	0.0499 (18)	−0.0045 (11)	−0.0147 (13)	0.0126 (13)
C54	0.0228 (13)	0.0306 (15)	0.0522 (19)	0.0005 (11)	−0.0021 (12)	−0.0164 (14)
C37	0.0356 (15)	0.0378 (16)	0.052 (2)	0.0042 (13)	−0.0268 (14)	−0.0145 (15)
C15	0.0433 (17)	0.0430 (17)	0.0359 (17)	−0.0180 (13)	0.0024 (13)	0.0013 (14)
C3	0.0372 (15)	0.0298 (15)	0.0447 (18)	−0.0035 (12)	−0.0125 (13)	0.0123 (13)
F2	0.134 (2)	0.178 (3)	0.198 (4)	−0.082 (2)	−0.138 (3)	0.120 (3)
F3	0.069 (4)	0.087 (4)	0.091 (3)	0.033 (3)	−0.017 (3)	−0.025 (3)
F4	0.078 (3)	0.081 (4)	0.087 (3)	−0.045 (3)	−0.030 (3)	0.016 (3)
F5	0.098 (4)	0.059 (3)	0.140 (6)	−0.039 (3)	−0.033 (4)	0.028 (4)
F6	0.084 (3)	0.177 (6)	0.066 (3)	0.029 (3)	−0.025 (2)	−0.066 (4)
F3A	0.076 (6)	0.165 (11)	0.127 (9)	−0.074 (7)	−0.066 (6)	0.091 (8)
F5A	0.37 (2)	0.117 (8)	0.126 (12)	−0.115 (13)	−0.064 (14)	−0.065 (8)
F6A	0.095 (6)	0.055 (5)	0.099 (6)	−0.012 (4)	0.027 (5)	0.030 (5)
F4A	0.086 (6)	0.050 (5)	0.113 (6)	0.029 (4)	−0.063 (5)	−0.026 (4)

### Geometric parameters (Å, °)

**Table:**

Co1—O2	1.8698 (16)	C10—H10A	0.9300
Co1—O1	1.8877 (14)	C28—H28A	0.9300
Co1—N2	1.911 (2)	C1—C2	1.381 (4)
Co1—N1	1.918 (2)	C1—H1A	0.9300
Co1—N3	1.927 (2)	C4—C3	1.386 (4)
Co1—N4	1.9442 (18)	C4—H4A	0.9300
Co2—O4	1.8759 (16)	C40—C39	1.478 (4)
Co2—O3	1.8921 (16)	C40—H40A	0.9700
Co2—N5	1.9225 (19)	C40—H40B	0.9700
Co2—N7	1.9234 (19)	C21—H21A	0.9300
Co2—N6	1.9217 (19)	C26—C25	1.361 (4)
Co2—N8	1.9477 (19)	C26—H26A	0.9300
N2—C7	1.350 (3)	C29—C30	1.384 (4)
N2—C11	1.350 (3)	C29—H29A	0.9300
O4—C48	1.347 (3)	C42—C43	1.378 (4)
O3—C47	1.352 (3)	C42—C41	1.380 (3)
O2—C20	1.339 (3)	C42—H42A	0.9300
N4—C18	1.493 (3)	C39—C38	1.380 (3)
N4—C12	1.500 (3)	C7—C8	1.369 (3)
N4—C6	1.501 (3)	C7—H7A	0.9300
N5—C29	1.342 (3)	C43—H43A	0.9300
N5—C33	1.350 (3)	C8—C9	1.387 (4)
N6—C35	1.340 (3)	C8—H8A	0.9300
N6—C39	1.352 (3)	C50—C55	1.413 (4)
N1—C1	1.336 (3)	C50—C51	1.414 (3)
N1—C5	1.352 (3)	C50—C49	1.425 (3)
O1—C19	1.349 (3)	C34—H34A	0.9700
N3—C13	1.339 (3)	C34—H34B	0.9700
N3—C17	1.355 (3)	C24—C23	1.369 (4)
N8—C34	1.490 (3)	C24—C25	1.406 (4)
N8—C46	1.498 (3)	C24—H24A	0.9300
N8—C40	1.503 (3)	C13—C14	1.378 (4)
C47—C56	1.374 (3)	C13—H13A	0.9300
C47—C48	1.428 (4)	C49—H49A	0.9300
N7—C41	1.341 (3)	C32—C31	1.378 (4)
N7—C45	1.348 (3)	C32—H32A	0.9300
C5—C4	1.372 (4)	C35—C36	1.379 (3)
C5—C6	1.497 (4)	C35—H35A	0.9300
C45—C44	1.377 (3)	C55—C54	1.422 (3)
C45—C46	1.499 (3)	C55—C56	1.426 (3)
C6—H6A	0.9700	C23—H23A	0.9300
C6—H6B	0.9700	C38—C37	1.375 (4)
C20—C21	1.363 (3)	C38—H38A	0.9300
C20—C19	1.442 (3)	C41—H41A	0.9300
C33—C32	1.378 (3)	O1W—H1WC	0.8500
C33—C34	1.506 (3)	O1W—H1WD	0.8500
C19—C28	1.371 (3)	C56—H56A	0.9300
C46—H46A	0.9700	C17—C16	1.377 (4)
C46—H46B	0.9700	C17—C18	1.499 (3)
C11—C10	1.373 (3)	C18—H18A	0.9700
C11—C12	1.493 (4)	C18—H18B	0.9700
P1—F5A	1.381 (10)	C36—C37	1.371 (4)
P1—F6A	1.416 (8)	C36—H36A	0.9300
P1—F3	1.460 (5)	C30—C31	1.381 (4)
P1—F5	1.519 (4)	C30—H30A	0.9300
P1—F2	1.560 (3)	C52—C51	1.373 (4)
P1—F1	1.571 (2)	C52—C53	1.392 (5)
P1—F4	1.648 (5)	C52—H52A	0.9300
P1—F6	1.658 (4)	C14—C15	1.381 (4)
P1—F4A	1.776 (9)	C14—H14A	0.9300
P1—F3A	1.797 (10)	C53—C54	1.369 (4)
P2—F12	1.587 (2)	C53—H53A	0.9300
P2—F10	1.581 (2)	C51—H51A	0.9300
P2—F7	1.5921 (19)	C16—C15	1.383 (4)
P2—F8	1.592 (2)	C16—H16A	0.9300
P2—F11	1.599 (2)	C31—H31A	0.9300
P2—F9	1.602 (2)	C2—C3	1.387 (4)
C44—C43	1.380 (4)	C2—H2A	0.9300
C44—H44A	0.9300	C12—H12A	0.9700
C27—C28	1.414 (3)	C12—H12B	0.9700
C27—C26	1.420 (3)	C25—H25A	0.9300
C27—C22	1.436 (3)	C9—H9A	0.9300
C48—C49	1.372 (3)	C54—H54A	0.9300
C22—C23	1.409 (3)	C37—H37A	0.9300
C22—C21	1.422 (3)	C15—H15A	0.9300
C10—C9	1.376 (4)	C3—H3A	0.9300
			
O2—Co1—O1	88.22 (6)	C43—C44—H44A	120.4
O2—Co1—N2	176.90 (7)	C45—C44—H44A	120.4
O1—Co1—N2	94.80 (7)	C28—C27—C26	122.9 (2)
O2—Co1—N1	89.65 (8)	C28—C27—C22	119.5 (2)
O1—Co1—N1	94.96 (8)	C26—C27—C22	117.6 (2)
N2—Co1—N1	89.39 (8)	O4—C48—C49	124.0 (2)
O2—Co1—N3	91.18 (8)	O4—C48—C47	116.0 (2)
O1—Co1—N3	95.57 (7)	C49—C48—C47	119.9 (2)
N2—Co1—N3	89.23 (8)	C23—C22—C21	122.4 (2)
N1—Co1—N3	169.46 (8)	C23—C22—C27	119.3 (2)
O2—Co1—N4	90.02 (7)	C21—C22—C27	118.3 (2)
O1—Co1—N4	178.21 (8)	C9—C10—C11	119.9 (2)
N2—Co1—N4	86.95 (8)	C9—C10—H10A	120.1
N1—Co1—N4	84.69 (8)	C11—C10—H10A	120.1
N3—Co1—N4	84.80 (8)	C19—C28—C27	121.3 (2)
O4—Co2—O3	88.14 (7)	C19—C28—H28A	119.4
O4—Co2—N5	94.45 (8)	C27—C28—H28A	119.4
O3—Co2—N5	89.98 (7)	N1—C1—C2	121.6 (2)
O4—Co2—N7	95.81 (8)	N1—C1—H1A	119.2
O3—Co2—N7	88.93 (7)	C2—C1—H1A	119.2
N5—Co2—N7	169.64 (8)	C5—C4—C3	119.0 (2)
O4—Co2—N6	92.82 (8)	C5—C4—H4A	120.5
O3—Co2—N6	175.86 (7)	C3—C4—H4A	120.5
N5—Co2—N6	93.96 (8)	C39—C40—N8	111.39 (19)
N7—Co2—N6	86.97 (8)	C39—C40—H40A	109.4
O4—Co2—N8	178.34 (8)	N8—C40—H40A	109.4
O3—Co2—N8	92.80 (7)	C39—C40—H40B	109.4
N5—Co2—N8	84.19 (8)	N8—C40—H40B	109.4
N7—Co2—N8	85.57 (8)	H40A—C40—H40B	108.0
N6—Co2—N8	86.34 (8)	C20—C21—C22	121.0 (2)
C7—N2—C11	119.4 (2)	C20—C21—H21A	119.5
C7—N2—Co1	125.87 (16)	C22—C21—H21A	119.5
C11—N2—Co1	114.74 (17)	C25—C26—C27	121.4 (2)
C48—O4—Co2	110.03 (15)	C25—C26—H26A	119.3
C47—O3—Co2	109.23 (15)	C27—C26—H26A	119.3
C20—O2—Co1	110.40 (14)	N5—C29—C30	121.4 (2)
C18—N4—C12	111.6 (2)	N5—C29—H29A	119.3
C18—N4—C6	112.28 (19)	C30—C29—H29A	119.3
C12—N4—C6	111.3 (2)	C43—C42—C41	119.3 (2)
C18—N4—Co1	106.17 (14)	C43—C42—H42A	120.4
C12—N4—Co1	110.25 (15)	C41—C42—H42A	120.4
C6—N4—Co1	104.94 (14)	N6—C39—C38	121.1 (3)
C29—N5—C33	119.7 (2)	N6—C39—C40	115.1 (2)
C29—N5—Co2	126.40 (17)	C38—C39—C40	123.7 (2)
C33—N5—Co2	113.92 (16)	N2—C7—C8	121.7 (2)
C35—N6—C39	119.3 (2)	N2—C7—H7A	119.2
C35—N6—Co2	124.98 (17)	C8—C7—H7A	119.2
C39—N6—Co2	114.29 (17)	C42—C43—C44	119.3 (2)
C1—N1—C5	119.9 (2)	C42—C43—H43A	120.4
C1—N1—Co1	126.58 (17)	C44—C43—H43A	120.4
C5—N1—Co1	113.30 (17)	C7—C8—C9	119.2 (2)
C19—O1—Co1	109.61 (13)	C7—C8—H8A	120.4
C13—N3—C17	119.1 (2)	C9—C8—H8A	120.4
C13—N3—Co1	127.29 (17)	C55—C50—C51	119.2 (2)
C17—N3—Co1	113.27 (15)	C55—C50—C49	119.4 (2)
C34—N8—C46	113.23 (19)	C51—C50—C49	121.4 (3)
C34—N8—C40	109.58 (18)	N8—C34—C33	107.6 (2)
C46—N8—C40	111.60 (17)	N8—C34—H34A	110.2
C34—N8—Co2	106.79 (13)	C33—C34—H34A	110.2
C46—N8—Co2	105.82 (13)	N8—C34—H34B	110.2
C40—N8—Co2	109.60 (15)	C33—C34—H34B	110.2
O3—C47—C56	123.2 (2)	H34A—C34—H34B	108.5
O3—C47—C48	116.3 (2)	C23—C24—C25	119.9 (3)
C56—C47—C48	120.4 (2)	C23—C24—H24A	120.1
C41—N7—C45	119.8 (2)	C25—C24—H24A	120.1
C41—N7—Co2	126.52 (18)	N3—C13—C14	121.8 (2)
C45—N7—Co2	113.41 (15)	N3—C13—H13A	119.1
N1—C5—C4	121.3 (2)	C14—C13—H13A	119.1
N1—C5—C6	113.9 (2)	C48—C49—C50	120.6 (3)
C4—C5—C6	124.9 (2)	C48—C49—H49A	119.7
N7—C45—C44	121.2 (2)	C50—C49—H49A	119.7
N7—C45—C46	114.00 (19)	C33—C32—C31	118.5 (3)
C44—C45—C46	124.8 (2)	C33—C32—H32A	120.7
C5—C6—N4	107.82 (19)	C31—C32—H32A	120.7
C5—C6—H6A	110.1	N6—C35—C36	121.7 (2)
N4—C6—H6A	110.1	N6—C35—H35A	119.2
C5—C6—H6B	110.1	C36—C35—H35A	119.2
N4—C6—H6B	110.1	C50—C55—C54	118.9 (2)
H6A—C6—H6B	108.5	C50—C55—C56	119.3 (2)
O2—C20—C21	123.2 (2)	C54—C55—C56	121.7 (3)
O2—C20—C19	116.0 (2)	C24—C23—C22	121.1 (3)
C21—C20—C19	120.7 (2)	C24—C23—H23A	119.4
N5—C33—C32	121.6 (2)	C22—C23—H23A	119.4
N5—C33—C34	114.2 (2)	C37—C38—C39	119.1 (3)
C32—C33—C34	124.2 (2)	C37—C38—H38A	120.4
O1—C19—C28	125.0 (2)	C39—C38—H38A	120.4
O1—C19—C20	115.75 (19)	N7—C41—C42	121.2 (2)
C28—C19—C20	119.2 (2)	N7—C41—H41A	119.4
N8—C46—C45	108.4 (2)	C42—C41—H41A	119.4
N8—C46—H46A	110.0	H1WC—O1W—H1WD	108.6
C45—C46—H46A	110.0	C47—C56—C55	120.2 (2)
N8—C46—H46B	110.0	C47—C56—H56A	119.9
C45—C46—H46B	110.0	C55—C56—H56A	119.9
H46A—C46—H46B	108.4	N3—C17—C16	121.7 (2)
N2—C11—C10	121.0 (2)	N3—C17—C18	113.7 (2)
N2—C11—C12	116.2 (2)	C16—C17—C18	124.5 (2)
C10—C11—C12	122.8 (2)	N4—C18—C17	107.3 (2)
F5A—P1—F6A	103.6 (9)	N4—C18—H18A	110.3
F5A—P1—F3	142.5 (8)	C17—C18—H18A	110.3
F6A—P1—F3	113.8 (4)	N4—C18—H18B	110.3
F5A—P1—F5	46.3 (7)	C17—C18—H18B	110.3
F6A—P1—F5	148.9 (5)	H18A—C18—H18B	108.5
F3—P1—F5	96.3 (3)	C37—C36—C35	119.0 (3)
F5A—P1—F2	91.6 (7)	C37—C36—H36A	120.5
F6A—P1—F2	100.7 (5)	C35—C36—H36A	120.5
F3—P1—F2	84.7 (3)	C29—C30—C31	118.5 (2)
F5—P1—F2	89.3 (3)	C29—C30—H30A	120.8
F5A—P1—F1	90.5 (7)	C31—C30—H30A	120.8
F6A—P1—F1	80.8 (5)	C51—C52—C53	120.4 (3)
F3—P1—F1	92.3 (3)	C51—C52—H52A	119.8
F5—P1—F1	90.6 (3)	C53—C52—H52A	119.8
F2—P1—F1	177.0 (2)	C13—C14—C15	119.1 (2)
F5A—P1—F4	128.3 (8)	C13—C14—H14A	120.5
F3—P1—F4	88.5 (3)	C15—C14—H14A	120.5
F5—P1—F4	171.5 (4)	C54—C53—C52	121.0 (3)
F2—P1—F4	84.1 (2)	C54—C53—H53A	119.5
F1—P1—F4	96.3 (2)	C52—C53—H53A	119.5
F6A—P1—F6	61.7 (4)	C52—C51—C50	120.4 (3)
F3—P1—F6	173.9 (3)	C52—C51—H51A	119.8
F5—P1—F6	88.8 (3)	C50—C51—H51A	119.8
F2—P1—F6	92.1 (3)	C17—C16—C15	118.7 (2)
F1—P1—F6	90.9 (2)	C17—C16—H16A	120.6
F4—P1—F6	86.0 (3)	C15—C16—H16A	120.6
F5A—P1—F4A	160.6 (7)	C32—C31—C30	120.3 (3)
F6A—P1—F4A	90.0 (5)	C32—C31—H31A	119.9
F5—P1—F4A	117.5 (4)	C30—C31—H31A	119.9
F2—P1—F4A	99.4 (3)	C3—C2—C1	118.6 (3)
F1—P1—F4A	77.9 (3)	C3—C2—H2A	120.7
F4—P1—F4A	69.1 (4)	C1—C2—H2A	120.7
F6—P1—F4A	151.1 (4)	C11—C12—N4	111.80 (19)
F5A—P1—F3A	88.4 (7)	C11—C12—H12A	109.3
F6A—P1—F3A	162.1 (6)	N4—C12—H12A	109.3
F2—P1—F3A	92.1 (3)	C11—C12—H12B	109.3
F1—P1—F3A	85.8 (3)	N4—C12—H12B	109.3
F4—P1—F3A	143.1 (5)	H12A—C12—H12B	107.9
F6—P1—F3A	130.9 (5)	C26—C25—C24	120.8 (2)
F4A—P1—F3A	75.5 (5)	C26—C25—H25A	119.6
F12—P2—F10	90.89 (14)	C24—C25—H25A	119.6
F12—P2—F7	89.80 (11)	C10—C9—C8	118.9 (2)
F10—P2—F7	179.22 (13)	C10—C9—H9A	120.6
F12—P2—F8	90.99 (13)	C8—C9—H9A	120.6
F10—P2—F8	90.03 (12)	C53—C54—C55	120.1 (3)
F7—P2—F8	90.32 (11)	C53—C54—H54A	120.0
F12—P2—F11	89.25 (13)	C55—C54—H54A	120.0
F10—P2—F11	89.88 (12)	C38—C37—C36	119.7 (2)
F7—P2—F11	89.77 (11)	C38—C37—H37A	120.2
F8—P2—F11	179.75 (13)	C36—C37—H37A	120.2
F12—P2—F9	178.54 (14)	C16—C15—C14	119.4 (3)
F10—P2—F9	89.12 (13)	C16—C15—H15A	120.3
F7—P2—F9	90.18 (12)	C14—C15—H15A	120.3
F8—P2—F9	90.47 (14)	C2—C3—C4	119.5 (3)
F11—P2—F9	89.30 (13)	C2—C3—H3A	120.3
C43—C44—C45	119.2 (2)	C4—C3—H3A	120.3
			
O1—Co1—N2—C7	−0.3 (2)	C34—N8—C46—C45	−154.22 (18)
N1—Co1—N2—C7	−95.2 (2)	C40—N8—C46—C45	81.6 (2)
N3—Co1—N2—C7	95.2 (2)	Co2—N8—C46—C45	−37.6 (2)
N4—Co1—N2—C7	−179.9 (2)	N7—C45—C46—N8	31.7 (3)
O1—Co1—N2—C11	177.81 (16)	C44—C45—C46—N8	−149.4 (2)
N1—Co1—N2—C11	82.88 (17)	C7—N2—C11—C10	−0.4 (3)
N3—Co1—N2—C11	−86.66 (17)	Co1—N2—C11—C10	−178.64 (19)
N4—Co1—N2—C11	−1.83 (17)	C7—N2—C11—C12	179.4 (2)
O3—Co2—O4—C48	−2.66 (14)	Co1—N2—C11—C12	1.1 (3)
N5—Co2—O4—C48	87.18 (15)	N7—C45—C44—C43	3.1 (4)
N7—Co2—O4—C48	−91.39 (15)	C46—C45—C44—C43	−175.7 (2)
N6—Co2—O4—C48	−178.63 (14)	Co2—O4—C48—C49	−174.19 (18)
O4—Co2—O3—C47	−0.52 (14)	Co2—O4—C48—C47	5.3 (2)
N5—Co2—O3—C47	−94.98 (14)	O3—C47—C48—O4	−6.1 (3)
N7—Co2—O3—C47	95.33 (14)	C56—C47—C48—O4	176.03 (19)
N8—Co2—O3—C47	−179.16 (14)	O3—C47—C48—C49	173.38 (19)
O1—Co1—O2—C20	−0.56 (15)	C56—C47—C48—C49	−4.5 (3)
N1—Co1—O2—C20	94.41 (15)	C28—C27—C22—C23	−176.2 (2)
N3—Co1—O2—C20	−96.10 (15)	C26—C27—C22—C23	1.5 (3)
N4—Co1—O2—C20	179.10 (15)	C28—C27—C22—C21	1.6 (3)
O2—Co1—N4—C18	61.73 (15)	C26—C27—C22—C21	179.4 (2)
N2—Co1—N4—C18	−118.95 (15)	N2—C11—C10—C9	0.8 (4)
N1—Co1—N4—C18	151.38 (16)	C12—C11—C10—C9	−179.0 (2)
N3—Co1—N4—C18	−29.45 (15)	O1—C19—C28—C27	177.9 (2)
O2—Co1—N4—C12	−177.29 (17)	C20—C19—C28—C27	0.1 (3)
N2—Co1—N4—C12	2.02 (16)	C26—C27—C28—C19	−178.3 (2)
N1—Co1—N4—C12	−87.65 (17)	C22—C27—C28—C19	−0.7 (3)
N3—Co1—N4—C12	91.53 (17)	C5—N1—C1—C2	−2.2 (4)
O2—Co1—N4—C6	−57.34 (16)	Co1—N1—C1—C2	−176.64 (18)
N2—Co1—N4—C6	121.98 (17)	N1—C5—C4—C3	−0.4 (4)
N1—Co1—N4—C6	32.31 (16)	C6—C5—C4—C3	179.6 (2)
N3—Co1—N4—C6	−148.52 (17)	C34—N8—C40—C39	132.3 (2)
O4—Co2—N5—C29	14.60 (19)	C46—N8—C40—C39	−101.5 (2)
O3—Co2—N5—C29	102.73 (19)	Co2—N8—C40—C39	15.4 (2)
N7—Co2—N5—C29	−173.4 (4)	O2—C20—C21—C22	−175.6 (2)
N6—Co2—N5—C29	−78.5 (2)	C19—C20—C21—C22	1.7 (3)
N8—Co2—N5—C29	−164.4 (2)	C23—C22—C21—C20	175.6 (2)
O4—Co2—N5—C33	−164.89 (16)	C27—C22—C21—C20	−2.2 (3)
O3—Co2—N5—C33	−76.76 (16)	C28—C27—C26—C25	176.7 (2)
N7—Co2—N5—C33	7.1 (5)	C22—C27—C26—C25	−1.0 (4)
N6—Co2—N5—C33	101.96 (17)	C33—N5—C29—C30	1.0 (3)
N8—Co2—N5—C33	16.06 (16)	Co2—N5—C29—C30	−178.50 (18)
O4—Co2—N6—C35	9.93 (19)	C35—N6—C39—C38	1.5 (3)
N5—Co2—N6—C35	104.59 (19)	Co2—N6—C39—C38	−165.65 (18)
N7—Co2—N6—C35	−85.75 (19)	C35—N6—C39—C40	−177.1 (2)
N8—Co2—N6—C35	−171.50 (19)	Co2—N6—C39—C40	15.8 (3)
O4—Co2—N6—C39	176.20 (16)	N8—C40—C39—N6	−20.6 (3)
N5—Co2—N6—C39	−89.14 (16)	N8—C40—C39—C38	160.9 (2)
N7—Co2—N6—C39	80.52 (16)	C11—N2—C7—C8	−0.4 (3)
N8—Co2—N6—C39	−5.23 (16)	Co1—N2—C7—C8	177.60 (18)
O2—Co1—N1—C1	−112.7 (2)	C41—C42—C43—C44	−2.6 (4)
O1—Co1—N1—C1	−24.5 (2)	C45—C44—C43—C42	−0.5 (4)
N2—Co1—N1—C1	70.3 (2)	N2—C7—C8—C9	0.8 (4)
N3—Co1—N1—C1	152.8 (4)	C46—N8—C34—C33	154.22 (18)
N4—Co1—N1—C1	157.3 (2)	C40—N8—C34—C33	−80.5 (2)
O2—Co1—N1—C5	72.53 (16)	Co2—N8—C34—C33	38.2 (2)
O1—Co1—N1—C5	160.71 (16)	N5—C33—C34—N8	−27.6 (3)
N2—Co1—N1—C5	−104.53 (17)	C32—C33—C34—N8	154.6 (2)
N3—Co1—N1—C5	−22.0 (5)	C17—N3—C13—C14	0.7 (4)
N4—Co1—N1—C5	−17.53 (17)	Co1—N3—C13—C14	173.82 (19)
O2—Co1—O1—C19	−0.01 (14)	O4—C48—C49—C50	−178.9 (2)
N2—Co1—O1—C19	−179.31 (14)	C47—C48—C49—C50	1.7 (3)
N1—Co1—O1—C19	−89.51 (15)	C55—C50—C49—C48	1.2 (3)
N3—Co1—O1—C19	91.00 (15)	C51—C50—C49—C48	−177.8 (2)
O2—Co1—N3—C13	108.6 (2)	N5—C33—C32—C31	−2.3 (4)
O1—Co1—N3—C13	20.3 (2)	C34—C33—C32—C31	175.3 (2)
N2—Co1—N3—C13	−74.5 (2)	C39—N6—C35—C36	−1.3 (3)
N1—Co1—N3—C13	−157.0 (4)	Co2—N6—C35—C36	164.35 (18)
N4—Co1—N3—C13	−161.5 (2)	C51—C50—C55—C54	0.9 (3)
O2—Co1—N3—C17	−77.94 (17)	C49—C50—C55—C54	−178.1 (2)
O1—Co1—N3—C17	−166.27 (17)	C51—C50—C55—C56	177.6 (2)
N2—Co1—N3—C17	98.98 (17)	C49—C50—C55—C56	−1.4 (3)
N1—Co1—N3—C17	16.5 (5)	C25—C24—C23—C22	−0.7 (4)
N4—Co1—N3—C17	11.97 (17)	C21—C22—C23—C24	−178.5 (2)
O3—Co2—N8—C34	59.34 (15)	C27—C22—C23—C24	−0.7 (4)
N5—Co2—N8—C34	−30.35 (15)	N6—C39—C38—C37	−0.7 (4)
N7—Co2—N8—C34	148.04 (15)	C40—C39—C38—C37	177.7 (2)
N6—Co2—N8—C34	−124.71 (15)	C45—N7—C41—C42	−0.7 (4)
O3—Co2—N8—C46	−61.57 (14)	Co2—N7—C41—C42	−174.20 (18)
N5—Co2—N8—C46	−151.26 (15)	C43—C42—C41—N7	3.3 (4)
N7—Co2—N8—C46	27.14 (14)	O3—C47—C56—C55	−173.45 (19)
N6—Co2—N8—C46	114.38 (14)	C48—C47—C56—C55	4.3 (3)
O3—Co2—N8—C40	177.96 (15)	C50—C55—C56—C47	−1.3 (3)
N5—Co2—N8—C40	88.27 (15)	C54—C55—C56—C47	175.3 (2)
N7—Co2—N8—C40	−93.33 (15)	C13—N3—C17—C16	1.8 (4)
N6—Co2—N8—C40	−6.09 (15)	Co1—N3—C17—C16	−172.2 (2)
Co2—O3—C47—C56	−178.64 (17)	C13—N3—C17—C18	−176.5 (2)
Co2—O3—C47—C48	3.6 (2)	Co1—N3—C17—C18	9.5 (3)
O4—Co2—N7—C41	−16.1 (2)	C12—N4—C18—C17	−79.8 (2)
O3—Co2—N7—C41	−104.1 (2)	C6—N4—C18—C17	154.48 (19)
N5—Co2—N7—C41	171.9 (4)	Co1—N4—C18—C17	40.3 (2)
N6—Co2—N7—C41	76.4 (2)	N3—C17—C18—N4	−33.4 (3)
N8—Co2—N7—C41	163.0 (2)	C16—C17—C18—N4	148.4 (3)
O4—Co2—N7—C45	170.03 (16)	N6—C35—C36—C37	0.4 (4)
O3—Co2—N7—C45	82.01 (16)	N5—C29—C30—C31	−2.2 (4)
N5—Co2—N7—C45	−2.0 (5)	N3—C13—C14—C15	−2.1 (4)
N6—Co2—N7—C45	−97.44 (16)	C51—C52—C53—C54	−0.4 (4)
N8—Co2—N7—C45	−10.88 (16)	C53—C52—C51—C50	1.1 (4)
C1—N1—C5—C4	1.7 (4)	C55—C50—C51—C52	−1.3 (3)
Co1—N1—C5—C4	176.90 (19)	C49—C50—C51—C52	177.6 (2)
C1—N1—C5—C6	−178.3 (2)	N3—C17—C16—C15	−2.8 (4)
Co1—N1—C5—C6	−3.1 (3)	C18—C17—C16—C15	175.3 (3)
C41—N7—C45—C44	−2.6 (3)	C33—C32—C31—C30	1.0 (4)
Co2—N7—C45—C44	171.76 (18)	C29—C30—C31—C32	1.1 (4)
C41—N7—C45—C46	176.4 (2)	N1—C1—C2—C3	1.2 (4)
Co2—N7—C45—C46	−9.3 (2)	N2—C11—C12—N4	0.5 (3)
N1—C5—C6—N4	29.7 (3)	C10—C11—C12—N4	−179.7 (2)
C4—C5—C6—N4	−150.3 (2)	C18—N4—C12—C11	115.8 (2)
C18—N4—C6—C5	−155.6 (2)	C6—N4—C12—C11	−117.9 (2)
C12—N4—C6—C5	78.5 (2)	Co1—N4—C12—C11	−1.9 (3)
Co1—N4—C6—C5	−40.8 (2)	C27—C26—C25—C24	−0.4 (4)
Co1—O2—C20—C21	178.46 (18)	C23—C24—C25—C26	1.3 (4)
Co1—O2—C20—C19	1.0 (2)	C11—C10—C9—C8	−0.4 (4)
C29—N5—C33—C32	1.3 (3)	C7—C8—C9—C10	−0.4 (4)
Co2—N5—C33—C32	−179.17 (18)	C52—C53—C54—C55	−0.1 (4)
C29—N5—C33—C34	−176.5 (2)	C50—C55—C54—C53	−0.2 (3)
Co2—N5—C33—C34	3.0 (3)	C56—C55—C54—C53	−176.8 (2)
Co1—O1—C19—C28	−177.24 (19)	C39—C38—C37—C36	−0.2 (4)
Co1—O1—C19—C20	0.6 (2)	C35—C36—C37—C38	0.4 (4)
O2—C20—C19—O1	−1.1 (3)	C17—C16—C15—C14	1.4 (5)
C21—C20—C19—O1	−178.6 (2)	C13—C14—C15—C16	1.0 (4)
O2—C20—C19—C28	176.9 (2)	C1—C2—C3—C4	0.2 (4)
C21—C20—C19—C28	−0.7 (3)	C5—C4—C3—C2	−0.6 (4)

### Hydrogen-bond geometry (Å, °)

**Table:**

*D*—H···*A*	*D*—H	H···*A*	*D*···*A*	*D*—H···*A*
O1W—H1WC···O3	0.85	1.97	2.808 (3)	170
O1W—H1WD···F4	0.85	2.11	2.940 (5)	165
O1W—H1WD···F4A	0.85	2.55	3.194 (11)	133
O1W—H1WD···F6A	0.85	2.30	3.142 (11)	171
